# Targeting thalamocortical circuits for closed-loop stimulation in Lennox–Gastaut syndrome

**DOI:** 10.1093/braincomms/fcae161

**Published:** 2024-05-07

**Authors:** Aaron E L Warren, Christopher R Butson, Matthew P Hook, Linda J Dalic, John S Archer, Emma Macdonald-Laurs, Frederic L W V J Schaper, Lauren A Hart, Hargunbir Singh, Lise Johnson, Katie L Bullinger, Robert E Gross, Martha J Morrell, John D Rolston

**Affiliations:** Department of Neurosurgery, Brigham and Women’s Hospital, Harvard Medical School, Boston, MA 02115, USA; Normal Fixel Institute for Neurological Diseases, University of Florida, Gainesville, FL 32608, USA; Normal Fixel Institute for Neurological Diseases, University of Florida, Gainesville, FL 32608, USA; University of Melbourne, Parkville, VIC 3052, Australia; Department of Neurology, Austin Health, Heidelberg, VIC 3084, Australia; University of Melbourne, Parkville, VIC 3052, Australia; Department of Neurology, Austin Health, Heidelberg, VIC 3084, Australia; University of Melbourne, Parkville, VIC 3052, Australia; Department of Neurology, Royal Children’s Hospital, Parkville, VIC 3052, Australia; Murdoch Children’s Research Institute, Parkville, VIC 3052, Australia; Department of Neurology, Brigham and Women’s Hospital, Harvard Medical School, Boston, MA 02115, USA; Department of Neurology, Brigham and Women’s Hospital, Harvard Medical School, Boston, MA 02115, USA; Department of Neurosurgery, Brigham and Women’s Hospital, Harvard Medical School, Boston, MA 02115, USA; NeuroPace, Mountain View, CA 94043, USA; Department of Neurology, Emory University Hospital, Atlanta, GA 30322, USA; Department of Neurosurgery, Emory University Hospital, Atlanta, GA 30322, USA; NeuroPace, Mountain View, CA 94043, USA; Department of Neurology and Neurological Science, Stanford University, Palo Alto, CA 94304, USA; Department of Neurosurgery, Brigham and Women’s Hospital, Harvard Medical School, Boston, MA 02115, USA

**Keywords:** epilepsy, Lennox–Gastaut syndrome, RNS, thalamus, MRI

## Abstract

This paper outlines the therapeutic rationale and neurosurgical targeting technique for bilateral, closed-loop, thalamocortical stimulation in Lennox–Gastaut syndrome, a severe form of childhood-onset epilepsy. Thalamic stimulation can be an effective treatment for Lennox–Gastaut syndrome, but complete seizure control is rarely achieved. Outcomes may be improved by stimulating areas beyond the thalamus, including cortex, but the optimal targets are unknown. We aimed to identify a cortical target by synthesizing prior neuroimaging studies, and to use this knowledge to advance a dual thalamic (centromedian) and cortical (frontal) approach for closed-loop stimulation. Multi-modal brain network maps from three group-level studies of Lennox–Gastaut syndrome were averaged to define the area of peak overlap: simultaneous EEG-functional MRI of generalized paroxysmal fast activity, [^18^F]fluorodeoxyglucose PET of cortical hypometabolism and diffusion MRI structural connectivity associated with clinical efficacy in a previous trial of thalamic deep brain stimulation. The resulting ‘hotspot’ was used as a seed in a normative functional MRI connectivity analysis to identify connected networks. Intracranial electrophysiology was reviewed in the first two trial patients undergoing bilateral implantations guided by this hotspot. Simultaneous recordings from cortex and thalamus were analysed for presence and synchrony of epileptiform activity. The peak overlap was in bilateral premotor cortex/caudal middle frontal gyrus. Functional connectivity of this hotspot revealed a distributed network of frontoparietal cortex resembling the diffuse abnormalities seen on EEG-functional MRI and PET. Intracranial electrophysiology showed characteristic epileptiform activity of Lennox–Gastaut syndrome in both the cortical hotspot and thalamus; most detected events occurred first in the cortex before appearing in the thalamus. Premotor frontal cortex shows peak involvement in Lennox–Gastaut syndrome and functional connectivity of this region resembles the wider epileptic brain network. Thus, it may be an optimal target for a range of neuromodulation therapies, including thalamocortical stimulation and emerging non-invasive treatments like focused ultrasound or transcranial magnetic stimulation. Compared to thalamus-only approaches, the addition of this cortical target may allow more rapid detections of seizures, more diverse stimulation paradigms and broader modulation of the epileptic network. A prospective, multi-centre trial of closed-loop thalamocortical stimulation for Lennox–Gastaut syndrome is currently underway.

## Teaser

Warren *et al*. outline the rationale and neurosurgical technique for a trial of bilateral, closed-loop, thalamocortical stimulation in Lennox–Gastaut syndrome, a severe epilepsy. A multi-modal synthesis of neuroimaging studies identified an epileptic ‘hotspot’ in premotor/frontal cortex. Electrophysiology is described in patients with neurostimulators targeting the centromedian nucleus and premotor cortex.

## Graphical Abstract

**Graphical Abstract:**
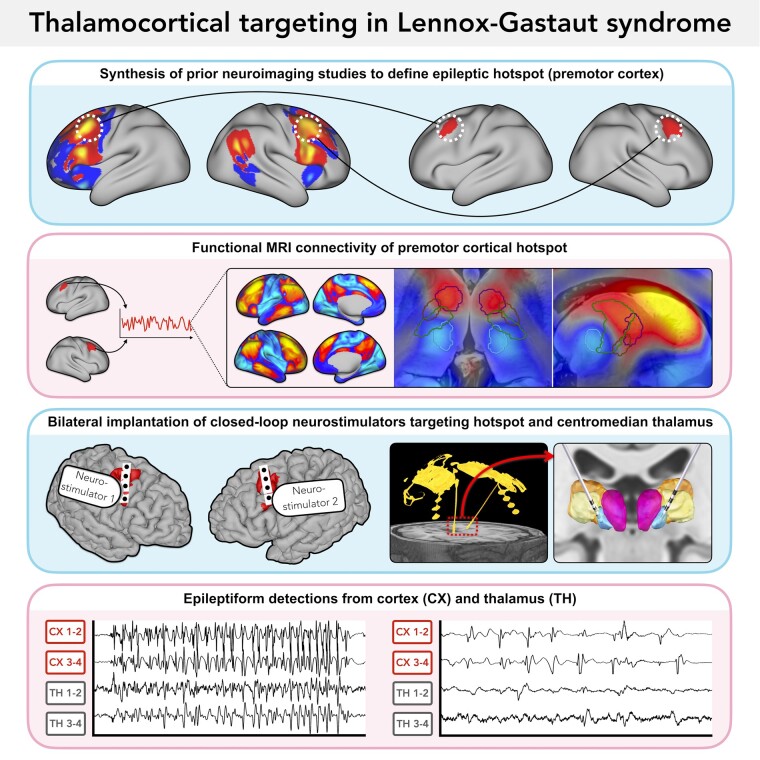

## Introduction

Lennox–Gastaut syndrome (LGS) is a childhood-onset epilepsy characterized by a collection of clinical and EEG features associated with severe disability and significant psychosocial impacts on families/caregivers.^1^ The syndrome develops secondarily to diverse aetiologies (e.g. genetic, structural and metabolic). Despite this, patients have similar seizure types (including tonic seizures, among others)^1^ and interictal epileptiform discharges [including generalized paroxysmal fast activity (GPFA) and slow spike-wave (SSW)] suggestive of shared neural mechanisms.

Knowledge of brain networks underlying LGS has advanced in recent years. Group-level studies using functional neuroimaging, including simultaneous EEG-fMRI and [^18^F]fluorodeoxyglucose PET (^18^F-FDG PET), have shown blood-oxygen-level-dependent (BOLD) changes during GPFA^2^ and SSW^3^ and altered glucose metabolism.^4^ There is also evidence that efficacy of thalamic deep brain stimulation (DBS) for LGS is dependent upon white-matter connections between the thalamus and brain areas implicated in GPFA.^5^ However, similarities between these findings, and their relationships to intrinsic brain circuits (e.g. motor pathways via which tonic seizures are expressed), have not been assessed.

These advances have occurred in parallel with rapid growth of neuromodulation treatments, including DBS,^6-12^ closed-loop/responsive neurostimulation (RNS System),^13,14^ hippocampal stimulation,^15-18^ focused ultrasound (FUS),^19^ transcranial magnetic stimulation (TMS),^20^ transcranial direct current stimulation (tDCS),^21^ chronic subthreshold cortical stimulation (CSCS)^22-24^ and implantable drug delivery devices.^25^ Closed-loop thalamocortical stimulation, targeting both thalamus and cortex, has also been proposed,^26^ seeking to record from and modulate seizure networks with greater coverage than thalamus-only approaches. These techniques are starting to be trialled in LGS, but their utility is hindered by ongoing uncertainty about optimal locations to target therapy.

Here we outline the therapeutic rationale and neurosurgical targeting strategy for a recently commenced multi-centre clinical trial of bilateral, closed-loop, combined thalamic and cortical stimulation for LGS. We aimed to localize a cortical target by synthesizing results from previous neuroimaging studies of LGS, and to map connectivity of this target using normative MRI.^27-30^ We hypothesized a discrete cortical location that may guide targeted neuromodulation and describe two patients who underwent RNS System implantations informed by these findings.

## Materials and methods

### Study design

This study comprises (i) a synthesis of prior neuroimaging studies in LGS, with comparison to connectivity features from open-access MRI in healthy adults^27-30^; and (ii) analysis of two patients with LGS who underwent RNS System implantations informed by (i). The prior studies were completed at Austin Health, Melbourne, with previously described ethical approvals.^2,4,5^ The surgical patients were recruited for the ‘RNS System LGS Feasibility Study’ (ClinicalTrials.gov ID = NCT05339126; IRB approval ID = 20221633), a prospective trial investigating preliminary safety and effectiveness of thalamocortical responsive neurostimulation in 20 patients with LGS. The two surgical patients included in the current analysis have a diagnosis of LGS consistent with the updated ILAE definition,^1^ including tonic seizures and at least one other seizure type, GPFA and SSW on EEG and intellectual disability.

### Synthesis of prior neuroimaging studies

**Figure 1:**
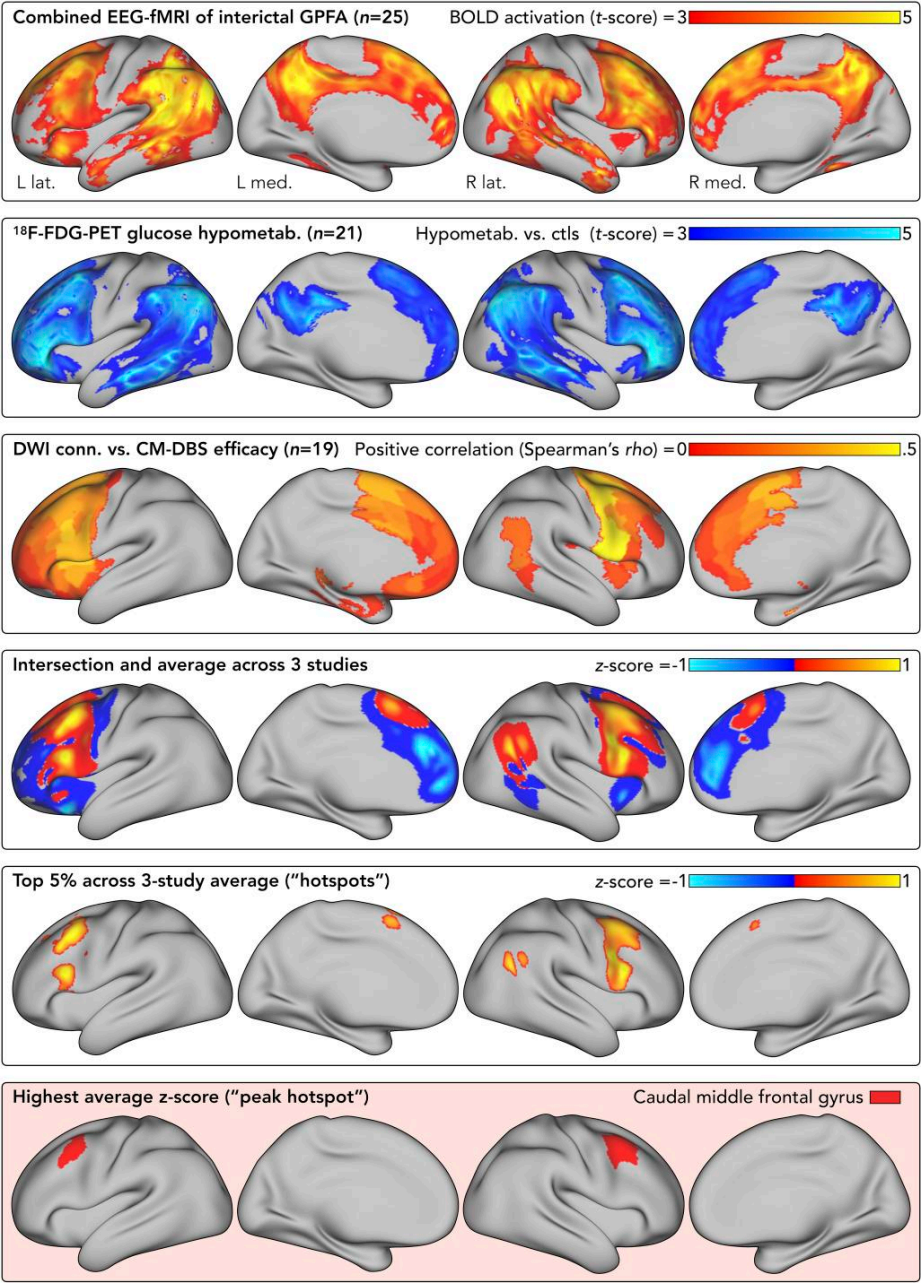
**Synthesis of brain network maps from neuroimaging studies of LGS.** Maps from three studies were combined to define a cortical hotspot: a combined EEG-fMRI study showing BOLD activation during GPFA discharges^2^ (first row), an ^18^F-FDG PET study showing areas of cortical hypometabolism^4^ (second row) and a DWI study showing areas where stronger connectivity with thalamic DBS sites was associated with greater clinical efficacy^5^ (third row). The statistical procedures used to derive these maps are described previously.^2,4,5^ For the current analysis, each of these maps was converted to *Z*-scores (to convert maps to a common unit of measurement) and the three *Z*-score maps were intersected and then averaged together (fourth row; positive values indicate areas of common greater involvement across the three studies, while negative values indicate areas of common lesser involvement). A top 5% threshold was applied to the average *Z*-score map to define cortical hotspots (fifth row). The area containing the highest average *Z*-score was in premotor cortex/caudal middle frontal gyrus (sixth row); this was deemed the ‘peak hotspot’ and used for subsequent analyses. Results are displayed on the Human Connectome Project (HCP) S900 *32k_fs_LR* inflated cortical surface (https://balsa.wustl.edu/QXj2). Cortical hotspot mapping results are available to download in fsaverage (surface) and MNI ICBM 2009b Asymmetric (volume) template spaces: https://osf.io/5bkec. Abbreviations: BOLD, blood-oxygen-level-dependent; CM-DBS, centromedian deep brain stimulation; Conn., connectivity; Ctls., controls; DWI, diffusion-weighted imaging; EEG-fMRI, electroencephalography-functional magnetic resonance imaging; ^18^F-FDG PET, ^18^F-fluorodeoxyglucose positron emission tomography; GPFA, generalized paroxysmal fast activity; Hypometab., hypometabolism; L, left; Lat, lateral; Med, medial; R, right.

We integrated maps from three neuroimaging studies of LGS (Fig. 1):

- EEG-fMRI^2^ was acquired in 25 patients with LGS (mean age = 22 years). Interictal GPFA was manually marked on in-scanner EEGs, with whole-brain statistical parametric mapping of individual-level BOLD changes. A group-level map (*t*-scores) of average BOLD activation was obtained.^2,5^
- ^18^F-FDG PET^4^ was compared between 21 patients with LGS (mean age = 15 years) and 18 controls (mean age = 19 years) with temporal lobe epilepsy (TLE), including only hemispheres without structural abnormalities and contralateral to the side of TLE. A group-level map (*t*-scores) of hypometabolism in LGS was computed.^4^ Note that this yielded a unilateral map; for the current analysis, we mirrored the map to become bilateral.
- Structural connectivity^5^ was assessed in 19 patients with LGS (mean age = 25 years) who participated in the ‘Electrical Stimulation of Thalamus for Epilepsy of Lennox-Gastaut phenotype’ (ESTEL) trial of bilateral thalamic centromedian DBS.^6^ Percentage seizure reductions (baseline to 3 months post-DBS) were correlated with normative and disease-matched diffusion MRI connectivity from DBS sites. The analysis was repeated across three seizure outcomes (diaries, ambulatory EEGs and a diary-EEG composite).^5^ This yielded 6 maps (Spearman correlations) i.e. 2 diffusion MRI datasets × 3 outcomes. For the current analysis, we averaged the six maps then thresholded to include only positive correlations (i.e. areas where stronger connectivity was associated with higher seizure reduction).

To identify hotspots, we (i) nonlinearly warped each map to a common volumetric brain template (MNI-ICBM009b nonlinear asymmetric template) using Advanced Normalization Tools (ANTs) software^31^; (ii) intersected maps to retain common non-zero voxels; (iii) converted each map to *Z*-scores; (iv) averaged the *Z*-score maps, such that *Z* > 0 indicates higher-than-average involvement across the studies; (v) to facilitate alignment with novel patient brains, projected the average *Z*-score map to a template cortical surface (FreeSurfer’s ‘fsaverage’)^32^; (vi) smoothed by 10 mm; and (vii) thresholded to show the top 5% of non-zero values (‘hotspots’).

**Figure 2:**
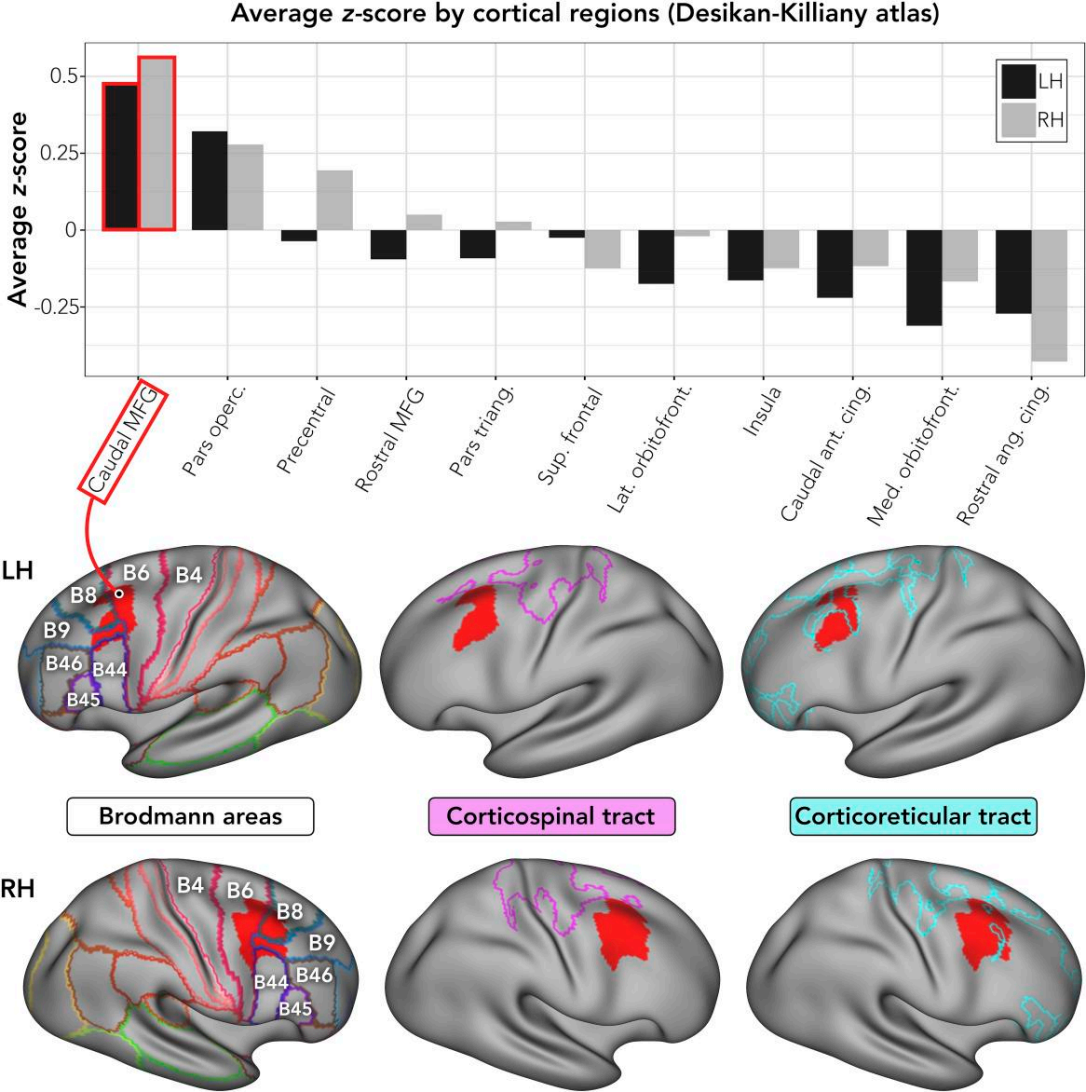
**Anatomical localization of the cortical hotspot and proximity to corticospinal and corticoreticular pathways.** To define the anatomical location containing the greatest convergence between the three prior neuroimaging studies of LGS, we calculated average *Z*-scores (contained in the map displayed in Fig. 1, fifth row) across anatomical parcels defined by the Desikan-Killiany atlas (https://surfer.nmr.mgh.harvard.edu/fswiki/CorticalParcellation), for left and right hemispheres separately. In both hemispheres, the highest average *Z*-score was in caudal middle frontal gyrus. Brain images show the hotspot’s position with respect to Brodmann areas and cortical projections of the corticospinal and corticoreticular tracts, as defined in a prior study of healthy controls (each tract is represented by an outline on the cortical surface; the area inside the outline indicates locations implicated in the tract; note that the corticospinal and corticoreticular tracts were derived using tractography that excluded contributions from the corticobulbar tract in ventral-lateral regions of frontal cortex).^29^ The hotspot showed greater overlap with the corticoreticular as opposed to corticospinal tract. Abbreviations: Ant., anterior; Cing., cingulate; Lat., lateral; LH, left hemisphere; Med., medial; MFG, middle frontal gyrus; Pars operc., pars opercularis; Pars triang., pars triangularis; RH, right hemisphere; Sup., superior.

This yielded a bilateral map of hotspots (Fig. 1). To stratify these by anatomical region and strength of involvement, we calculated the mean *Z*-score (across non-zero vertices) within each cortical area of the Desikan-Killiany atlas.^32^ For each hemisphere, the hotspot within the cortical area showing highest mean *Z*-score was deemed the ‘peak hotspot’ (Fig. 2). We chose to use the Desikan-Killiany atlas because it is an anatomical atlas based on major gyral and sulcal boundaries, which suits the intended neurosurgical application of our findings.

### Normative connectivity

**Figure 3:**
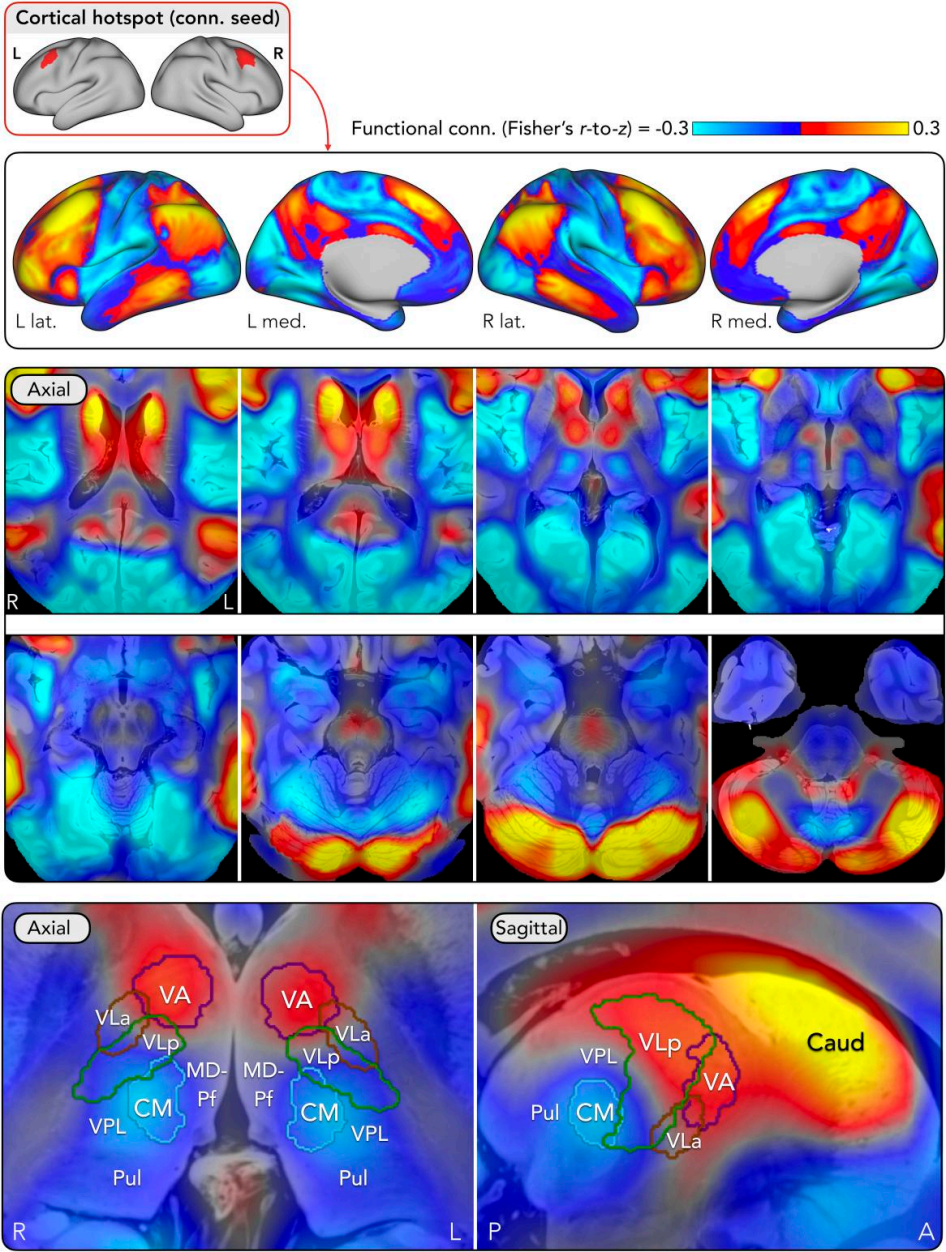
**Normative functional connectivity showing brain networks connected to the cortical hotspot.** A binary bilateral mask of the cortical hotspot (first row) was used as a seed in a whole-brain functional connectivity analysis employing a normative dataset of resting-state fMRI from 1000 healthy adults.^28^ Functional connectivity was computed using Fisher’s *r*-to-*Z* transformed Pearson correlation coefficients. Cortical (second row) and subcortical (third and fourth rows) regions showing positive and negative functional connectivity with the hotspot are displayed. A zoomed-in view of the thalamus is shown (fourth row) with nuclei overlaid as outlines (as defined by the THOMAS atlas).^33^ Subcortical connectivity is displayed upon a 7 tesla MRI scan of the *ex vivo* human brain (available from: https://doi.org/10.1038/s41597-019-0254-8).^34^ Abbreviations: A, anterior; Caud, caudate; CM, centromedian; Conn., connectivity; L, left; Lat, lateral; Med, medial; MD-Pf, mediodorsal-parafascicular; P, posterior; Pul, pulvinar; VA, ventral anterior; VLa, ventral lateral anterior; VLp, ventral lateral posterior; VPL, ventral posterolateral; R, right.

A normative approach^30^ was used to investigate functional connectivity of the peak cortical hotspot. The purpose of this analysis was to understand the intrinsic networks in which the hotspot is embedded to infer its potential influence upon other brain areas, both pathologically (e.g. potential propagation pathways of epileptiform activity involving the hotspot) and therapeutically (e.g. remote brain areas that may be influenced by neuromodulation of the hotspot). A whole-brain connectivity analysis was performed using resting-state fMRI from 1000 healthy adults.^27,28^ The fMRI data and pre-processing pipeline are publicly available.^27,28^ For each participant, the mean BOLD time course within a bilateral mask of the hotspot was used as a seed, and pair-wise Fisher’s *r*-to-*Z* transformed Pearson correlations were calculated with every brain voxel. Results were averaged across participants, yielding a map of the hotspot’s functional connections (Fig. 3).

Using the same process, we also explored functional connectivity of the thalamic stimulation target, the centromedian nucleus, using a bilateral mask of this nucleus obtained from the Thalamus Optimized Multi Atlas Segmentation (THOMAS) atlas.^33^ The purpose of this analysis was to compare connectivity of the cortical hotspot target with that of the centromedian thalamic target.

### Proximity to motor pathways

Seizures involving prominent motor manifestations, including tonic seizures of LGS, likely engage motor pathways in the cortex, brainstem and spinal cord, but the specific pathways involved are incompletely understood. To address this, we compared the hotspot to recent mappings of the human corticospinal and corticoreticular pathways derived from diffusion MRI tractography^29^ (Fig. 2). We reasoned that this would permit inference of motor tracts likely involved in LGS, including those that may transmit seizure activity or mediate treatment efficacy.

### Thalamocortical implantation

The hotspot guided electrode positioning in two patients with LGS (aged 37 and 22 years; females) who underwent bilateral RNS System implantation of frontal cortex and thalamic centromedian nucleus as part of the ‘RNS System LGS Feasibility Study’. At the time of writing, neither patient was receiving active stimulation due to a 3-month post-implantation baseline period in the study where devices were programmed to detect and record epileptiform activity only.

**Figure 4:**
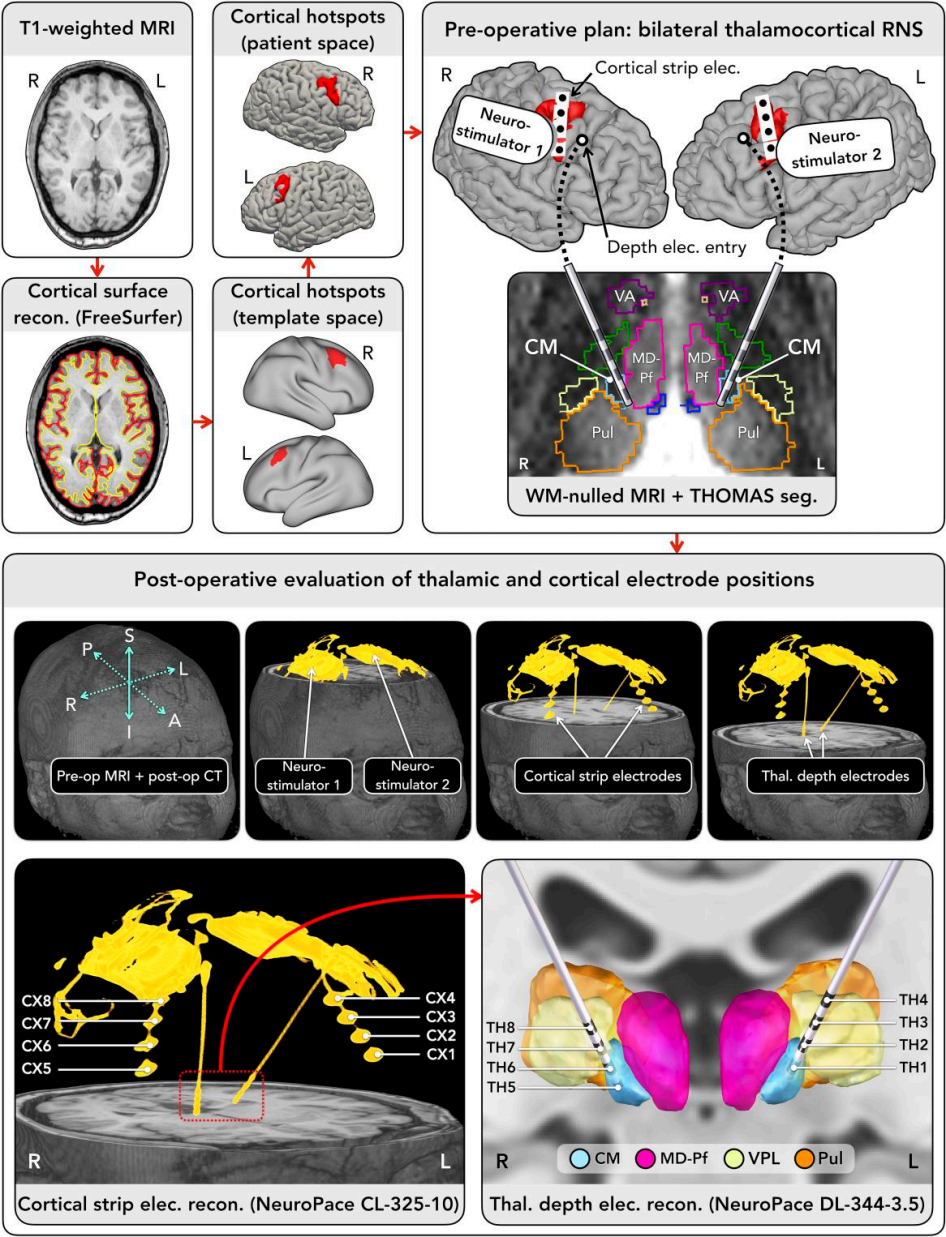
**Preoperative plan for bilateral thalamocortical RNS System implantation and postoperative evaluation of electrode positions.** The preoperative plan involved warping the location of the cortical hotspot from a template brain space (FreeSurfer’s ‘fsaverage’; https://surfer.nmr.mgh.harvard.edu/fswiki/FsAverage)^32^ to each patient’s native brain space. The location of the hotspot in native patient brain space was used to guide placement of the cortical strip lead on each brain side. Thalamic depth lead positions were planned using direct targeting of the centromedian nucleus, as seen on each patient’s white-matter-nulled MRI scan,^35^ aided by an atlas-based segmentation of the thalamus performed using THOMAS software.^33^ Entry points for the thalamic depth leads were typically located anterior to the cortical leads. Postoperative CT co-registered to the preoperative MRI was used to evaluate electrode positions. Abbreviations: A, anterior; CL, cortical lead; CM, centromedian; CX, cortex; DL, depth lead; I, inferior; L, left; MD-Pf, mediodorsal-parafascicular; MRI, magnetic resonance imaging; P, posterior; Pre-op, preoperative; Post-op, postoperative; Pul, pulvinar; R, right; Recon., reconstruction; RNS, responsive neurostimulation; Seg., segmentation; S, superior; TH, thalamus; Thal., thalamus; THOMAS, Thalamus Optimized Multi Atlas Segmentation; VA, ventral anterior; VPL, ventral posterolateral; WM, white matter.

Each patient was implanted with two NeuroPace devices (model RNS-320), one per hemisphere (Fig. 4). Each neurostimulator was connected to a cortical strip lead (model CL-325-10; four contacts, 10 mm spacing) implanted over the hotspot, and a depth lead (model DL-344-3.5; four contacts, 3.5 mm spacing) implanted into the thalamus. The neurostimulators operated independently (i.e. they detected/recorded epileptiform activity from each hemisphere separately).

Surgical planning used 3T MRI with T_1_-weighted magnetization-prepared-rapid-gradient-echo (MPRAGE; 1 mm^3^) and ‘white-matter nulled’ (WM-nulled)^35^ MPRAGE (1 mm^3^) scans. The hotspot defined here in ‘fsaverage’ template space was warped to each patient’s anatomy and visualized on the pial surface to guide cortical lead placement. Direct targeting^36^ of the centromedian nucleus was performed using WM-nulled MRI, aided by an atlas-based segmentation using THOMAS software.^33^ Lead positions were assessed by co-registering postoperative CT to preoperative MRI. Cortical leads were assessed for proximity to the hotspot, while thalamic leads were reconstructed in Lead-DBS^37^ and assessed for proximity to adjacent nuclei.^33^

**Figure 5:**
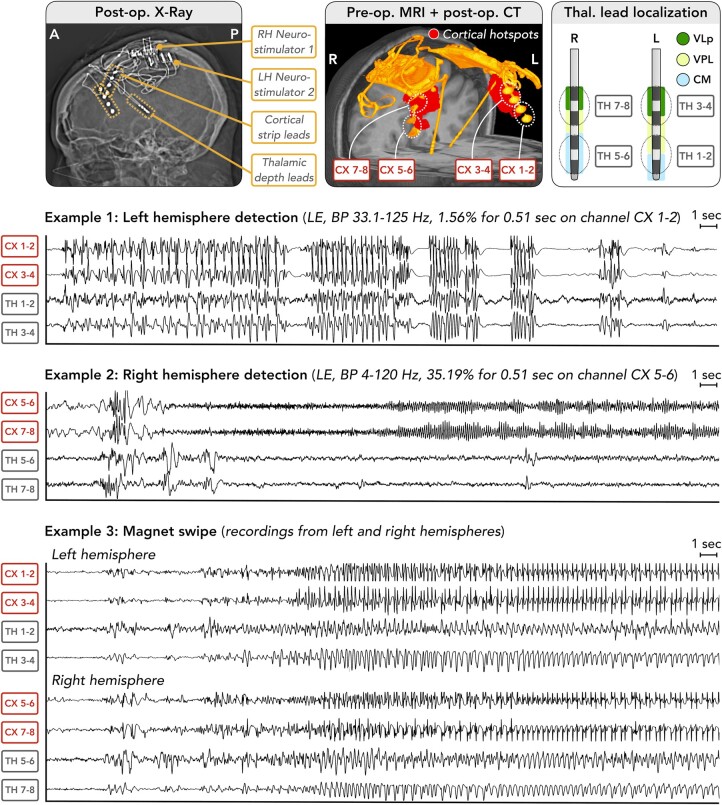
**Thalamocortical electrophysiology in Patient 1.** A female patient with LGS aged in her 30 s underwent bilateral thalamocortical RNS System implantation as outlined in Fig. 4. Postoperative CT co-registered to preoperative MRI was used to evaluate cortical and thalamic lead positions, including positions within thalamic nuclei defined by the THOMAS atlas^33^ (first row). Simultaneous recordings from the cortical hotspot and thalamus are shown for three example events in a bipolar montage (each example is 20 s in duration; note that recordings are displayed following a *Z*-score transformation to yield similar scaling of amplitudes across channels; this was performed due to the lower amplitude of signals recorded from the thalamus compared to the cortex): (i) a long episode detection from the left hemisphere, showing synchronous runs of ∼3–4 Hz spike-wave and ∼8–10 Hz polyspike discharges in both cortex and thalamus, with intermixed low-voltage fast activity in thalamic channel 1–2 (detection settings: bandpass detector on cortical channel 1–2 with minimum frequency of 33.1 Hz, maximum frequency of 125 Hz, minimum amplitude of 1.56% and minimum duration of 0.51 s); (ii) a long episode detection from the right hemisphere, showing a burst of ∼8–10 Hz polyspike activity in both cortex and thalamus, followed by low-voltage fast activity seen earlier and at higher frequency in the cortex than thalamus, which then evolves into higher amplitude alpha activity, again showing higher frequency in cortical channels (detection settings: bandpass detector on cortical channel 5–6 with minimum frequency of 4 Hz, maximum frequency of 120 Hz, minimum amplitude of 35.19% and minimum duration of 0.51 s); and (iii) a patient/caregiver magnet swipe across both left and right devices, yielding a bilateral recording showing apparently synchronous (between cortex and thalamus, and likely between left and right hemispheres) polyspike/low-voltage fast activity evolving to 3–4 Hz spike-and-wave discharges. Abbreviations: A, anterior; BP, bandpass; CM, centromedian; CT, computed tomography; CX, cortex; L, left; LH, left hemisphere; LE, long episode; MRI, magnetic resonance imaging; P, posterior; Pre-op., preoperative; Post-op., postoperative; R, right; RH, right hemisphere; TH, thalamus; Thal., thalamic; VLp, ventral lateral posterior; VPL, ventral posterolateral.

**Figure 6:**
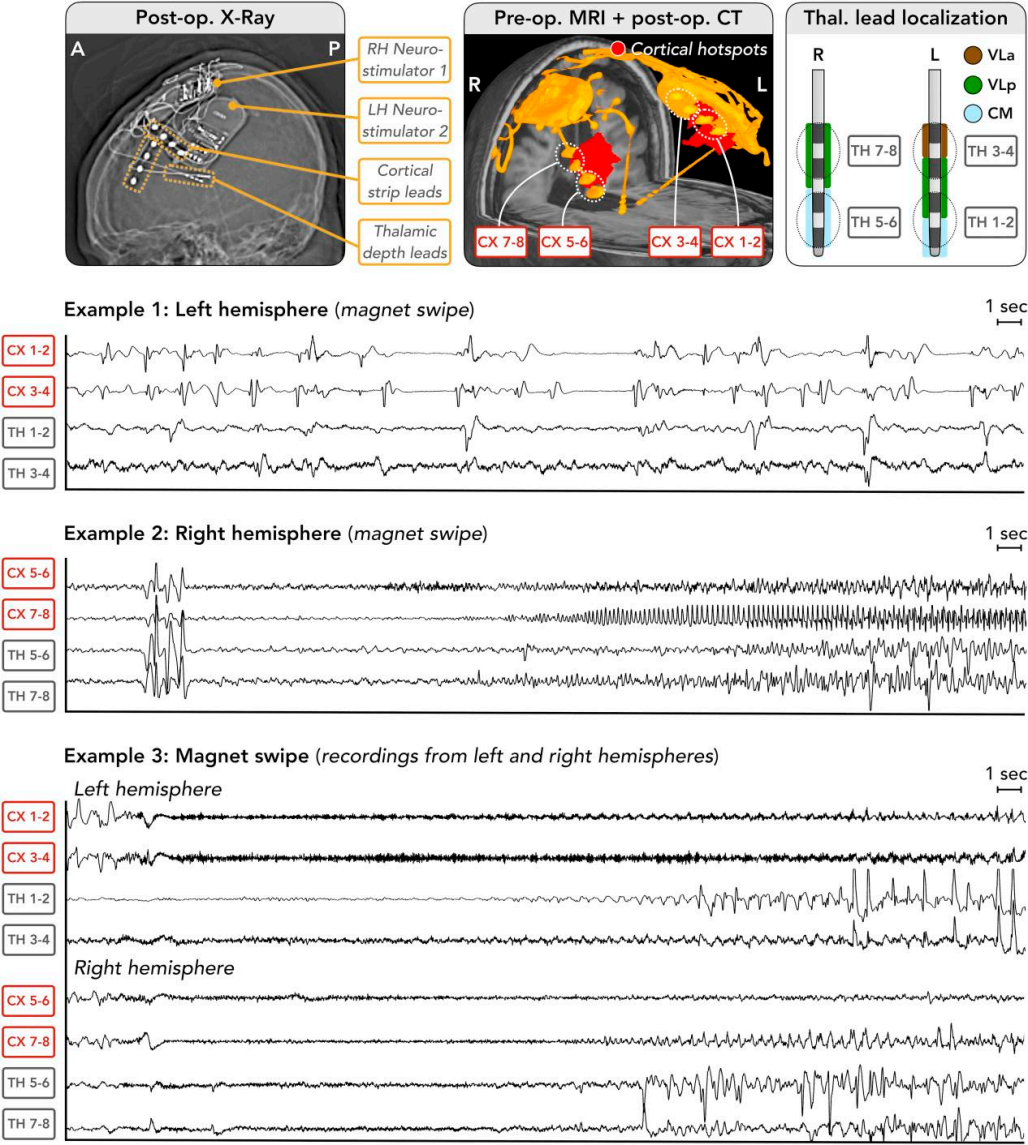
**Thalamocortical electrophysiology in Patient 2.** A female patient with LGS aged in her 20 s underwent bilateral thalamocortical RNS System implantation; other details as per Figs 4 and 5. Three examples are shown: (i) a patient/caregiver magnet swipe across the left hemisphere device only, showing a run of fluctuating slow (∼1–1.5 Hz) spike-and-wave discharges typical of the interictal EEG in LGS, being more sustained and showing earlier temporal onset in cortical relative to thalamic channels; (ii) a magnet swipe across the right hemisphere device only, showing a brief burst of sharps with synchronous onset in cortical and thalamic channels, followed by rhythmic theta in all electrodes and then low-voltage fast activity seen earliest in cortical channel 5–6. Distinct patterns of evolution are then seen in the cortical compared to thalamic channels; and (iii) a magnet swipe across both left and right devices, yielding a bilateral recording of rhythmic sharps transitioning to evolving low-voltage fast activity more prominently observed in cortical channels, then delayed onset of high-amplitude spike discharges in the thalamus. Abbreviations: A, anterior; CM, centromedian; CT, computed tomography; CX, cortex; L, left; LH, left hemisphere; MRI, magnetic resonance imaging; P, posterior; Pre-op., preoperative; Post-op., postoperative; R, right; RH, right hemisphere; TH, thalamus; Thal., thalamic; VLa, ventral lateral anterior; VLp, ventral lateral posterior.

Detection settings were updated periodically to ensure adequate event detection. Example events described here were detected using bandpass filters (see Figs 5 and 6 for description). Additional events were recorded as ‘magnet swipes’,^13^ in which patients/caregivers swipe a magnetic wand over the device when they witness a seizure, triggering a recording. These produced bilateral recordings within ∼1–2 s of each other when the magnet was swiped over both devices; this delay was in part due to the time taken for the magnet to be moved from one side of the head to the other. Events were reviewed for similarity to EEG signatures of LGS.

We also assessed relative temporal onsets of detected events between cortex and thalamus. For each patient, we extracted 10 detections per RNS System device (i.e. 20 recordings per patient; 10 each from the left and right devices). Events were selected using an electrographic seizure classifier^38^ specifically developed for RNS System recordings. For each recording, the classifier outputs the probability that it contains an electrographic seizure on each lead; from these, we extracted the top 10 recordings with highest probability of having a seizure on both leads. The patients’ treating physician (author K.L.B.), a board-certified epileptologist, visually reviewed each recording to determine whether onset occurred first in the cortical lead, first in the thalamic lead, or simultaneously in cortical and thalamic leads. An event was deemed to occur first in the cortical (or thalamic) lead if the onset could be seen at least 0.25 s prior to onset in the thalamic (or cortical) lead. If onset times could not be distinguished by at least 0.25 s, the event was deemed to occur ‘simultaneously’ in both leads. A threshold of 0.25 s was used as this was the minimum difference in onsets that could be clearly distinguished by eye.

### Statistical analysis

*Z*-scores were used for the synthesis of statistical brain maps from prior neuroimaging studies (Figs 1 and 2). Fisher’s *r*-to-*Z* transformed Pearson correlation coefficient was used to compute normative fMRI functional connectivity (Fig. 3 and Supplementary Fig. 1).

## Results

### Cortical hotspot and normative connectivity

Hotspot areas (top 5% *Z*-scores) were in bilateral frontal cortex anterior to precentral sulcus, including caudal superior, middle and inferior frontal gyri, as well as right inferior parietal lobule (Fig. 1). Other areas of lesser involvement (outside top 5%) included bilateral anterior insula, anterior cingulate and medial prefrontal cortex, and right posterior temporal cortex. Areas of non-involvement mainly included primary motor/sensory cortex, posterior insula and occipital lobe.

In both hemispheres, the peak hotspot (i.e. within the cortical area showing highest mean *Z*-score) was in premotor cortex/caudal middle frontal gyrus (MFG) covering portions of Brodmann areas 6 and 8 and the posterior aspect of area 9 (Fig. 2). The peak hotspot overlapped with cortical projections of the corticoreticular tract, but not corticospinal projections (Fig. 2).

Normative connectivity revealed a bilateral network (Fig. 3) closely resembling the distributed areas of epileptic involvement identified by EEG-fMRI and ^18^F-FDG PET (Fig. 1). Strongest positive connectivity was seen with MFG, inferior parietal lobule, posterolateral temporal, precuneus, anterior insula and dorsolateral prefrontal cortex. In contrast, negative connectivity was seen with pericentral, posterior insula, anterior temporal and occipital cortex. Subcortically, positive connectivity included posterior (Crus I/II) and ventral medial (lobule IX) cerebellum, dorsal pons, caudate and ventral anterior, ventral lateral and anterior thalamic nuclei, while negative connectivity was seen with hippocampus, amygdala, caudal putamen, globus pallidus and ventral anterior cerebellum (lobule VIIIb). Interestingly, positive connectivity was not seen with the centromedian nucleus; rather, negative connectivity was seen there—i.e. BOLD signals in premotor cortex and centromedian nucleus tended to counter-fluctuate.

A distinct pattern of connectivity was seen when seeding from the centromedian nucleus (Supplementary Fig. 1). Positive functional connectivity was observed with pericentral, occipital, insula, anterior cingulate, supplementary motor and anterior temporal cortex, and subcortically with the putamen, globus pallidus, midbrain, pons and dorsal medial (lobules I–VI) and ventral lateral (lobules VIIIa and VIIIb) cerebellum. Negative connectivity was seen with MFG (including the cortical hotspot location), superior frontal gyrus, inferior parietal lobule, posterolateral temporal, precuneus and medial prefrontal cortex, and subcortically with the posterior (Crus I/II) and ventral medial (lobule IX) cerebellum.

### Intracranial electrophysiology

Example epileptiform event recordings are in Figs 5 and 6. A variety of event types were observed, reflecting characteristic EEG signatures of LGS (e.g. low-voltage fast activity, polyspike and SSW). Magnet swipes across both devices confirmed that clinically evident seizures had an electrographic pattern that was bilateral and most likely synchronous between hemispheres, accounting for a ∼1–2 s temporal delay between the two recordings caused by the time taken to move the magnet from one side of the head to the other.

Event onset times were assessed in 40 recordings with highest probability of containing an electrographic seizure on both cortical and thalamic leads. 23/40 (57.5%) of recordings had onset first in the cortical lead, with a median 1.5 s delay before onset in the thalamic lead; 16/40 (40%) had simultaneous onsets in cortical and thalamic leads; and the onset times in 1/40 (2.5%) could not be determined due to the recording being continuously epileptiform throughout. No recordings showed an onset first in the thalamus.

## Discussion

Neuromodulation is rapidly changing the treatment landscape for LGS. Thalamic targets have been trialled, including DBS of the centromedian nucleus.^6-9^ However, adequate seizure control remains elusive: many patients who undergo DBS continue to experience a seizure burden that far exceeds that of other epilepsy syndromes, with families/caregivers sometimes reporting that the practical benefits are limited (e.g. patients still require round-the-clock supervision).^6^ Further benefits may be gained by exploring targets beyond the thalamus; however, until recently, the cortical substrates of LGS were ill-defined, in part due to the diffuse expression of epileptic activity in this syndrome. Here we identify a premotor cortical hotspot and apply this knowledge to advance a closed-loop thalamocortical stimulation approach. In doing so, we establish a targeting strategy that may be transferrable to other neuromodulation therapies for LGS, and potentially other diseases of thalamocortical origin.

### Convergent evidence for a premotor hotspot

These results represent a convergence of three distinct neuroimaging modalities, from nearly a decade of research into the epileptic network of LGS. First, EEG-fMRI measured BOLD changes during GPFA,^2,3^ revealing bilateral activation of frontal and parietal association cortex. This pattern was similar between children and adults,^2^ and across diverse aetiologies of LGS,^2,3^ suggesting it is a shared mode of reaction to the epileptogenic insult—and thus may also be a shared target of treatment or biomarker of treatment response, as recently demonstrated.^5,39^

Second, ^18^F-FDG PET assessed glucose hypometabolism,^4^ another marker of epileptogenic tissue. Patients with LGS showed extensive hypometabolism that closely reflected the cortical pattern of BOLD activation during GPFA,^2,3^ again implicating mainly frontal and parietal association cortex.

Third, diffusion MRI examined white-matter connections yielding higher seizure reduction in the ESTEL trial of centromedian DBS.^5,6^ In addition to subcortical connections (including brainstem, cerebellum, and putamen), clinical efficacy was most positively correlated with connectivity between stimulation sites and areas of premotor and caudal prefrontal cortex.^5^ In contrast, efficacy was less or negatively correlated with connectivity to parietal, occipital, and temporal lobes. This frontal emphasis agrees with SPECT observations showing that the early phase of tonic seizures involves increased blood flow frontally, but not later phases.^40^

Across these studies, the area of strongest convergence was in premotor cortex, most prominently the caudal MFG encompassing Brodmann areas 6 and 8 and the caudal aspect of Brodmann area 9. Anatomically, this region sits between dorsolateral prefrontal cortex anteriorly (Brodmann areas 9 and 46) and primary motor cortex/precentral gyrus posteriorly (Brodmann area 4).

Normative fMRI of this hotspot revealed a bilateral network of positive connectivity resembling the distributed cortical patterns of EEG-fMRI activation and ^18^F-FDG PET hypometabolism. This suggests the broader epileptic network of LGS manifests via intrinsic brain circuitry and can be reproduced from correlated activity of a single node within this network. Thus, modulation of the hotspot may influence the wider areas showing abnormal function in LGS, akin to the distributed effects of focal lesions upon the networks in which they are located.^41^

However, a less anticipated finding was the pattern of connectivity seen in the thalamus. The hotspot showed positive connectivity with ventral anterior, ventral lateral, and anterior nuclei, whereas it showed negative connectivity with the centromedian nucleus.

The centromedian nucleus is currently the only thalamic stimulation target for which there is emerging evidence of efficacy in LGS.^6-9^ The observation of negative connectivity between the cortical hotspot and centromedian nucleus suggests the two areas may be located in distinct functional networks,^30^ or their BOLD signals may be phase-delayed and separated by greater path length,^42^ at least within the normative fMRI dataset analysed here.^27,28^ Whether the same holds true in LGS will be important to explore in future studies; it is possible that patients’ connectivity deviates from controls.

This raises several hypotheses about the efficacy mechanisms underlying centromedian stimulation. One possibility is that it ‘disengages’ the premotor hotspot-related network (visualized in Fig. 3) and engages the centromedian-related network (visualized in Supplementary Fig. 1), thereby reducing time spent in a brain state that appears more vulnerable to seizures. This is supported by reports of increased arousal during centromedian DBS,^6,43^ suggesting a shift away from a state associated with more frequent seizures in LGS—i.e. reduced arousal, for example, during sleep.^44^

A second possibility is that the sign of functional connectivity may hint at whether excitatory or inhibitory stimulation is likely to be effective. Using the same normative fMRI dataset employed in our study, Fox *et al*.^30^ explored connectivity between cortical and subcortical sites where TMS and DBS respectively show efficacy, in each of several diseases. Cortical sites where inhibitory TMS was beneficial tended to show positive connectivity with the DBS site, whereas those where excitatory TMS was beneficial tended to be negatively connected.^30^ Thus, our finding of negative connectivity may indicate a role for divergent stimulation paradigms when targeting the two areas (i.e. inhibitory stimulation of the cortical hotspot and excitatory stimulation of the centromedian nucleus, or vice versa).

A third possibility is that stimulation of and/or seizure detection from thalamic areas beyond, or additional to, the centromedian nucleus may be required to achieve optimal effects. Indeed, analysis of stimulation ‘sweet-spots’ in the ESTEL trial suggested that the optimal DBS location extended anterolaterally from the centromedian nucleus, into the adjacent ventral lateral nucleus (posterior subdivision)^5^—noting that it was these connections with the sweet-spot, not connections with the CM *per se*, that contributed to our definition of the premotor cortical hotspot in the current study. Similarly, a recent case series of thalamic responsive neurostimulation in idiopathic generalized epilepsy suggested that seizure detection may be more sensitive outside the centromedian nucleus.^14^ A potential implication of these findings is that the posterior subdivision of the ventral lateral nucleus (also termed the ventral intermediate nucleus, or Vim) may be a more effective stimulation target for LGS. Interestingly, a recent case study of Vim-DBS demonstrated seizure control in a patient with focal epilepsy.^45^

However, these findings require further elaboration considering the small number of studies, limited sampling of thalamic stimulation sites, absence of efficacy comparisons between different targets and ongoing debate concerning interpretations of negative fMRI connectivity. It is also unknown whether different symptoms of LGS are best addressed via the same or distinct targets; our hotspot mapping was biased towards GPFA, hypometabolism and the seizure outcomes recorded in ESTEL (which did not specifically measure atypical absence seizures, for example, due to challenges in documenting this seizure type in LGS).^6^ Indeed, there is some evidence that different brain networks underlie different epileptiform features of LGS, including distinct networks associated with GPFA and SSW.^3^

### Corticoreticular–reticulospinal involvement

The precise role of premotor cortex and related pathways in LGS remains uncertain. However, some links can be drawn with tonic seizures,^46^ the most characteristic seizure type in these patients and a therapeutic priority given their potential to cause serious injury and ‘drop attacks.’ They involve sustained (∼10–20 s) and typically symmetric increases in muscle tone, mostly affecting truncal and proximal muscle groups that normally support postural adjustments and balance.^47^ On scalp EEG, they are accompanied by diffuse, 10–25 Hz fast activity, often accentuated over frontal channels.

At first glance, tonic seizures may seem better explained by an origin in (or stronger involvement of) primary motor cortex as opposed to premotor cortex, the former being a main source of volitional motor output in primates via the corticospinal pathway. However, our findings implicate the latter, raising several interpretations stemming from the functions and connections of premotor cortex, and—of potential relevance to the aetiological diversity of LGS—its pattern of similar, and at times maladaptive, neuroplastic response to various brain insults.^48^

**Figure 7:**
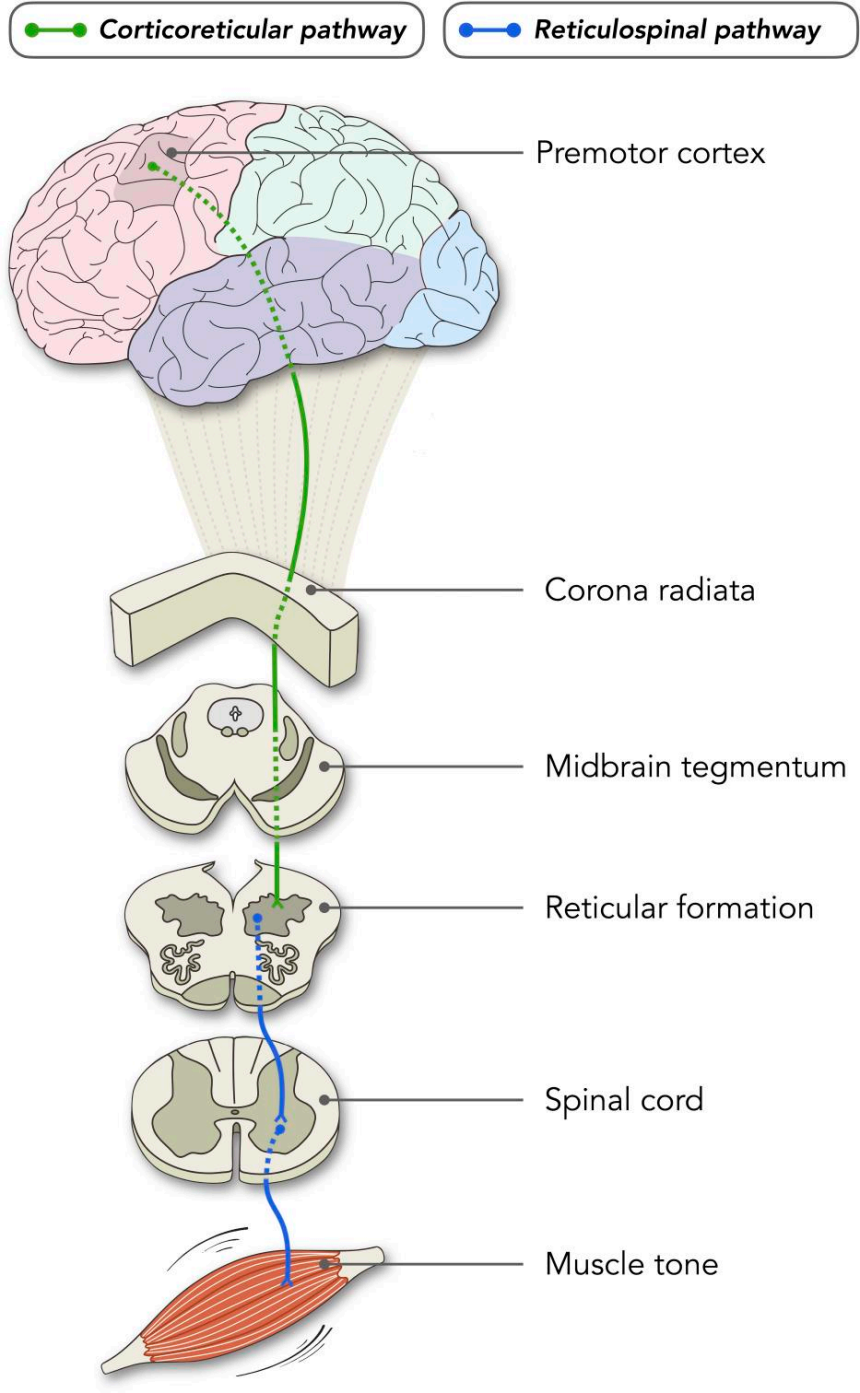
**Schematic showing the corticoreticular–reticulospinal pathway postulated to be involved in tonic seizures of LGS.** The cortical hotspot in premotor cortex/caudal middle frontal gyrus is located in areas from which the ‘extrapyramidal’ corticoreticular tract projects from the cortex, via the corona radiata and midbrain tegmentum, to the reticular formation of the brainstem. These brainstem terminations co-locate with reticulospinal tracts that descend to the spinal cord and regulate truncal and proximal muscle tone, among other functions.

In humans,^29^ a lesser-studied motor pathway can be traced from our observed hotspot to the musculature implicated in tonic seizures. Specifically, premotor cortex includes projections of the ‘extrapyramidal’ corticoreticular tract (Fig. 7), which traverses the corona radiata, internal capsule and midbrain tegmentum before terminating in the pontomedullary reticular formation.^29^ These terminations co-locate with reticulospinal tracts that descend from brainstem to spinal cord and regulate truncal and proximal muscle tone, among other functions.^49^

There is burgeoning interest in the role of the corticoreticular–reticulospinal pathway in motor recovery and neuroplastic reorganization following brain injuries,^48,50,51^ including stroke.^52,53^ For example, patients with stroke who recover walking ability after unilateral damage of the corticospinal tract show higher MRI white-matter fibre volume in the contralateral corticoreticular tract compared to those who do not recover.^52^ These changes are thought to be compensatory, where the extrapyramidal system is ‘upregulated’ to partially assume functions previously supported by damaged pathways.^50,52^

However, this upregulation may not always be beneficial, particularly in the immature brain. Spasticity has been linked to abnormally high corticoreticular–reticulospinal excitability in stroke^53^ and cerebral palsy.^54^ There is emerging evidence that such aberrant strengthening of motor pathways is age-dependent, with greater upregulation seen when injuries occur earlier in life,^55^ coinciding with windows of increased excitability during typical neurodevelopment.

Applying these concepts to LGS, it is tempting to imagine a similar maladaptive process whereby epileptogenic insults in the developing brain drive abnormal upregulation of corticoreticular–reticulospinal excitability, laying the groundwork for a generalized seizure network superimposed upon the extrapyramidal system. This hypothesis is supported by the recent observation of abnormally ‘increased’ myelination seen early after seizure onset in a rodent model of generalized epilepsy, and that prompt pharmacological blockade of maladaptive myelination can abrogate epilepsy progression.^56^ However, further experimental studies are needed to confirm the role of the corticoreticular–reticulospinal pathway in LGS.

### Clinical utility

Our mapping of a cortical hotspot has potential applications beyond the trial of thalamocortical responsive neurostimulation described here. For example, it may be the basis for pilot studies of non-invasive therapies for LGS, like TMS,^20^ FUS,^19^ tDCS,^21^ or CSCS,^22-24^ which have shown potential effectiveness when delivered to similar ‘hotspots’ in other epilepsies (e.g. FUS of hippocampus in TLE).^19^ It may also guide the placement of emerging ‘sub-scalp’ seizure monitoring devices,^57^ or inform the design of animal models of LGS^58^ that more accurately recapitulate the human phenotype by targeting brain circuits like the ones we identified here.

Intracranial recordings confirm that characteristic epileptiform features of LGS can be detected from the cortical hotspot, and that their onsets most commonly precede the thalamus, consistent with previous observations.^59^ We anticipate that these cortical implants will allow more precise detection and stimulation strategies, and modulation of more areas within the epileptic network underlying LGS, than thalamus-only approaches. For example, cortical detections that precede the thalamus may permit more rapid delivery of stimulation (either to the cortex, thalamus, or both), while those that evolve independently of the thalamus may avoid seizures going undetected. Another possibility is the delivery of divergent or asynchronous stimulation paradigms, allowing therapy to be tailored to different patterns of abnormal activity occurring in the cortex and thalamus.

### Limitations and future directions

Our hotspot mapping does not imply this is the only region with abnormal function in LGS; the epileptic network of LGS is more diffuse (Fig. 1). Our goal in mapping this hotspot was not to define the extent of epileptic involvement, but rather to guide selection of a discrete stimulation/detection target, given that diffuse intracranial coverage of all involved cortical areas is infeasible.

Additionally, the results represent a convergence between group-level studies, but the same hotspot may not be seen in every individual. Indeed, although the epileptic network of LGS is similar between distinct aetiologies,^2^ individual differences are also seen. For example, EEG-fMRI patterns can variably show activation of patient-specific cortical lesions in addition to the recognized group-level pattern.^3^ We anticipate that individual patient brain mapping may improve neuromodulation efficacy for LGS, like how group-level targets are individualized in other disease contexts.^60^

## Supplementary Material

fcae161_Supplementary_Data

## Data Availability

Cortical hotspot mapping results are available to download in fsaverage (surface) and MNI ICBM 2009b Asymmetric (volume) template spaces: https://osf.io/5bkec.
