# Supplementary material for: Targeting thalamocortical circuits for closed-loop stimulation in Lennox–Gastaut syndrome
Source: Brain Commun. 2024 May 7;6(3):fcae161. doi: 10.1093/braincomms/fcae161 (PMC11099664; doi:10.1093/braincomms/fcae161)
Supplement: fcae161_Supplementary_Data [file fcae161_supplementary_data.pdf]

Supplementary Material

Targeting thalamocortical circuits for closed-loop stimulation in Lennox-Gastaut syndrome

Corresponding author

Aaron E.L. Warrren

Department of Neurosurgery, Brigham and Women's Hospital, Harvard Medical School

Hale Building for Transformative Medicine, 60 Fenwood Road, Boston, MA, USA

awarren15@bwh.harvard.edu

Contents

- Supplementary Figure 1: Normative functional connectivity of the thalamic centromedian nucleus
- Supplementary References

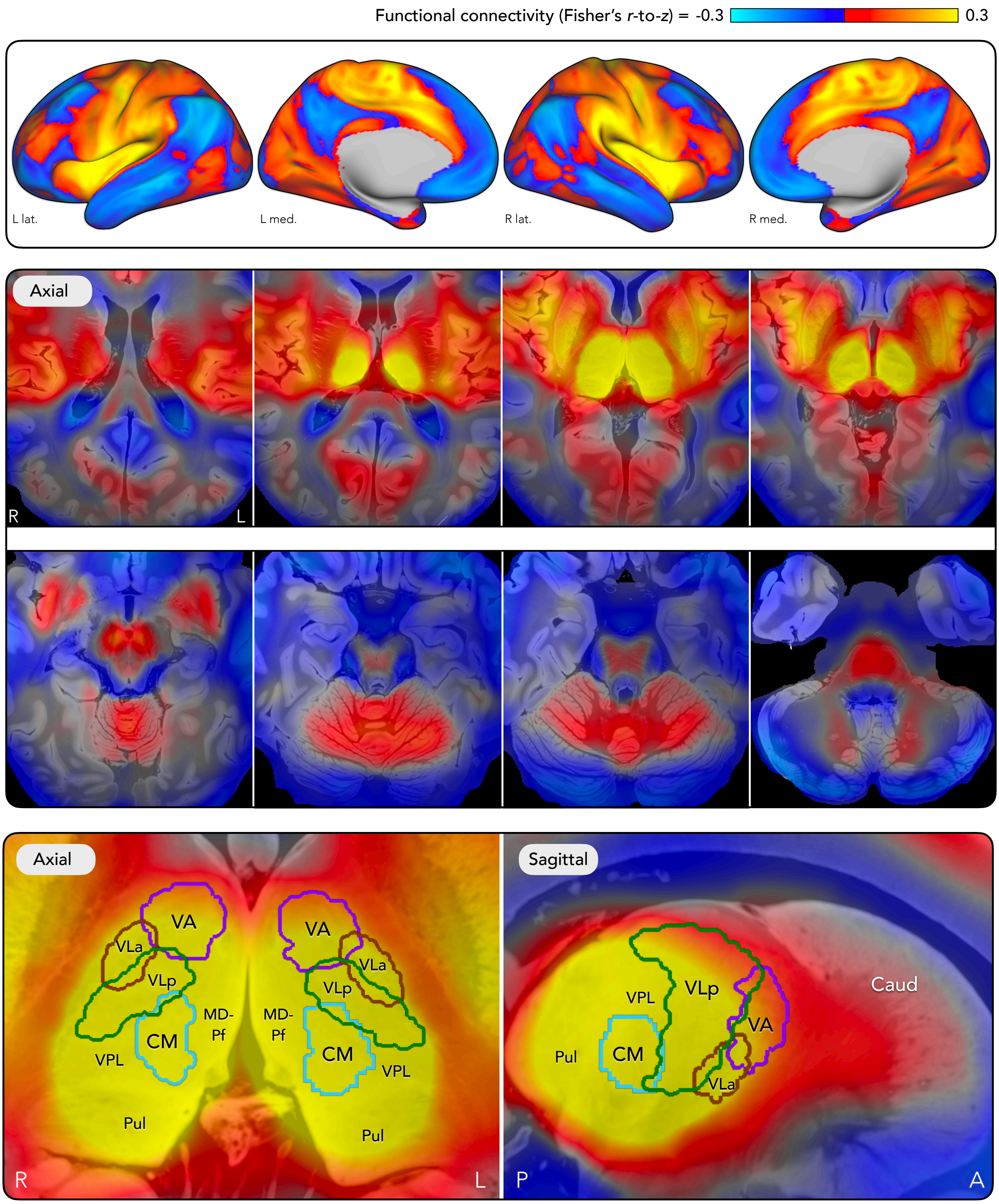

Supplementary Figure 1: Normative functional connectivity of the thalamic centromedian nucleus. A binary, bilateral mask of the thalamic centromedian nucleus (obtained from the Thalamus Optimized Multi Atlas Segmentation [THOMAS] atlas)[1] was used as a seed in a whole-brain functional connectivity analysis employing a normative dataset of resting-state functional MRI from 1,000 healthy adults.[2] Functional connectivity

was computed using Fisher's *r*-to-*z* transformed Pearson correlation coefficients. Cortical (first row) and subcortical (second and third rows) regions showing functional connectivity with the centromedian nucleus are displayed in orange/yellow colors (indicating positive connectivity) or blue/light blue colors (indicating negative or "anti-correlated" connectivity). A close-up view of the thalamus is shown (third row) with nuclei overlaid as colored outlines (as defined by the THOMAS atlas).[1] Subcortical connectivity is displayed upon a 7 Tesla MRI scan of the *ex vivo* human brain (available from: https://doi.org/10.1038/s41597-019-0254-8).[3] ***Abbreviations:*** A, anterior; Caud, caudate; CM, centromedian; Lat, lateral; L, left; Med, medial; MD-Pf, mediodorsal-parafascicular; P, posterior; Pul, pulvinar; VA, ventral anterior; VLa, ventral lateral anterior; VLp, ventral lateral posterior; VPL, ventral posterolateral; R, right.

SUPPLEMENTARY REFERENCES

1. Su, JH, Thomas, FT, Kasoff, WS, et al. Thalamus Optimized Multi Atlas Segmentation (THOMAS): fast, fully automated segmentation of thalamic nuclei from structural MRI. Neuroimage 2019;194:272-282. DOI: https://doi.org/10.1016/j.neuroimage.2019.03.021
2. Cohen, A, Soussand, L, McManus, P, Fox, Michael. GSP1000 Preprocessed Connectome. Harvard Dataverse, V3. DOI: https://doi.org/10.7910/DVN/ILXIKS
3. Edlow, BL, Mareyam, A, Horn, A, et al. 7 Telsa MRI of the ex vivo human brain at 100 micron resolution. Sci Data 2019;6(1):244. DOI: https://doi.org/10.1038/s41597-019-0254-8
